# Patent Landscape Analysis of Dental Caries in Primary Teeth

**DOI:** 10.3390/ijerph16122220

**Published:** 2019-06-24

**Authors:** Zsuzsa Bencze, Nadine Fraihat, Orsolya Varga

**Affiliations:** Department of Preventive Medicine, Faculty of Public Health, University of Debrecen, Debrecen 4002, POB 400, Hungary; nadine.fraihat@sph.unideb.hu (N.F.); varga.orsolya@sph.unideb.hu (O.V.)

**Keywords:** childhood caries, patent landscape, primary teeth, innovation trends

## Abstract

Inventions from the field of health research are mostly protected by patents. The main objective of this study is to identify the research and development trends of dental innovations for children, with a special focus on the inventions for dental caries in primary teeth and early childhood caries (ECC) by performing a patent landscape analysis on a global scale with special attention to the role of European countries in patenting activities. A patent landscape analysis is a tool used to identify trends in different areas of innovations. Patents and patent applications were extracted from Orbit Intelligence. The keyword based search process was refined by manual selection and grouped into prevention, treatment and diagnosis categories. The absolute number and legal status of patent families, priority years, priority countries, and assignees were examined. The total number of patents of dental caries in primary teeth was 61. According to the legal status of the patents, 27% are granted, 19% pending and 54% are dead. The earliest patent is from 1931 and the most recent is from 2018. Regarding the field of inventions, 37 patents were identified as prevention, 16 patents were treatment and 8 were diagnostics related. China holds the most patents. The huge burden of dental caries in primary teeth is poorly represented in global research and development. Additionally, inventions in dental caries of the primary dentition from the European Union lagged far behind China and the US, highlighting our insufficient research initiatives and programs.

## 1. Introduction

Although it is largely preventable, dental caries remains one of the most common chronic diseases of early childhood [1]. It is five times more common than asthma and seven times more common than hay fever among children [2]. The health care system is confronted with handling the extreme consequences of primary teeth caries in hospital emergency departments and operating rooms [3]. Caries of the deciduous teeth also predispose a child towards increased caries risk in their permanent dentition [4], and remains largely untreated in children [5], though it is highly preventable through early detection [6]. Inventions and innovative techniques might aid the caries prevention and management process.

Today inventions from the field of health research are mostly protected by patents. A patent gives the right to the patent holders to exclude others from making, using, selling, and importing the invention for a limited period of time, usually twenty years. The most recent information on a technological field is generally found in patent documents, and many of the databases provide free access to 30 million patent documents from around the world [7]. The recent broad access to such databases and their user-friendly visualization facilitate the use of patent landscaping to identify major development technical and uncertain areas and, consequently may facilitate future research and development (R&D) strategies.

The main objective of this study is to identify the research and development trends of dental innovations for children, with a special focus on the inventions for dental caries in primary teeth and early childhood caries (ECC) by performing a patent landscape analysis on a global scale with special attention to the role of European countries in the patenting activity.

By definition, ECC is the presence of 1 or more decayed (having noncavitated or cavitated lesions), missing (due to caries), or filled tooth surface in any primary tooth in a child 71 months of age or younger [8]. The disease can begin as early as the teeth begin to emerge and often progresses rapidly [9]. ECC is a multifactorial disease that develops from the interaction of different factors, such as cariogenic microorganisms (e.g., bacteria, primarily Streptococcus mutans), exposure to fermentable carbohydrates, and an unfavorable social background [10]. The period of ECC poses specific dilemmas that differentiate it from later periods of childhood caries, including challenges with breastfeeding during the night or swallowing toothpaste on a regular basis. Thus, early childhood period requires special attention and specific procedures both in diagnosis and in treatment to successfully deal with caries. The consequences of ECC often include a higher risk of new carious lesions in both the primary and the permanent teeth, hospitalizations, high treatment costs, loss of school days, and they also negatively affect the oral health-related quality of life [11]. Typically, ECC requires extensive dental repair, often in an operating room under general anesthesia. When left untreated, it can destroy the child’s teeth, and have a strong, lasting effect on a child’s general health status.

## 2. Materials and Methods

In this study, patents and patent applications were extracted from Orbit Intelligence [7]. Questel is a leading licensor of intellectual property-related data, offering over 250 patent, design and trademark authorities in bibliographic form, 30 patent authorities in full-text form with translations that allow English language searching across the entire database. The patents were searched combining different search terms related to deciduous teeth and caries. Keywords of the disease were obtained from the Medical Subject Headings (MeSH) database of the National Library of Medicine, which uses a vocabulary thesaurus for indexing articles for PubMed and the Orbit keyword search wizard. The final set of keywords was: (dental caries OR tooth decay OR dental cavity OR teeth decay OR caries OR decay OR cavity OR cariosity)/TI/AB/IW/CLMS AND (milk tooth OR deciduous tooth OR baby tooth OR child tooth OR primary tooth OR deciduous tooth OR milk teeth OR deciduous teeth OR baby teeth OR child teeth OR primary teeth OR deciduous teeth OR children teeth)/TI/AB/IW/CLMS. Truncation to cover different endings, singular/plural etc. was also used. The search was not limited by date, classification code or geographical region globally. Searches were carried out in the titles, abstracts, claims of patent documents and were refined by manual selection. We selected all the patents for analysis, which were related to caries of deciduous teeth in a broad sense, from a very early age to children still having primary teeth.

Duplicates were removed by creating simple families which represent the family members of a particular patent record with same priority dates. To simplify the trend analysis, we have grouped the patents according to the function of the inventions into three categories: prevention, treatment and diagnostics. We also classified these inventions according to their field of application: closely related to management of caries in primary teeth (specific patents) and not closely related ones (generic patents).

## 3. Results

### 3.1. Patenting Trends

The total number of patents related to dental innovations for children was 61 (see the list in Appendix A). The selected patents were examined with age specificity because some of the inventions may be intended for use in adults. 39% of the selected patents were particularly related to children under the age of 6 (e.g., baby oral hygiene tools, baby cookies). 43% of the patents were related to children and primary teeth with no age specified or specified for age of 6 and above (e.g., children toothpaste, cyst treatment of the primary dentition, school dental screening tool), and 18% of the inventions were not exclusively intended for children, but for use by both children and adults (e.g., pain management, dental matrix, teledental system).

43 patents (70.5%) were specifically related to caries prevention, diagnostics and treatment, while 18 patents were more generic, not closely related to caries management of primary teeth. We would like to underline that even if some patents indirectly address dental caries in primary teeth, they may have an important role in children’s oral health in the form of providing screening support, predicting delay in teeth development, early orthodontic appliances, or inventions to inhibit bad habits.

**Figure 1:**
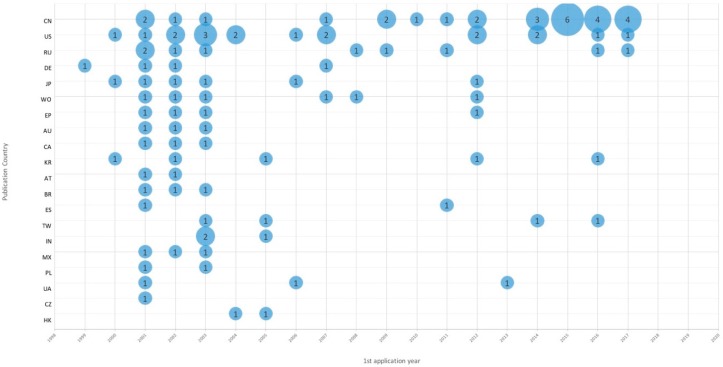
Publication country by year. This graph represents the location of patent publications and their changes over time. Abbreviations: CN-China, US-United States, RU-Russia, DE-Germany, JP-Japan, WO-World Intellectual Property Organization, EP-European, AU-Australia, CA-Canada, KR-Korea, Republic of, AT-Austria, BR-Brazil, ES-Spain, TW-Taiwan, IN-India, MX-Mexico, PL-Poland, UA-Ukraine, CZ-Czech Republic, HK-Hong Kong. The size of the blue circles represents the number of the patent documents.

The patents were grouped by invention-based families (FamPat), so a family is considered to be granted when at least one member of the family is a granted patent. If there is no granted member in the family, then the status is pending. According to their legal status, 27% of the patents are granted, 19% of them are pending and 54% of the patents are dead (patents that are no longer in-force as they are lapsed, revoked or expired). The first patent was filed in 1931 but patenting activity became intense in the 1990s, with the most innovative year being 2001 with 18 patents. The majority of patent families have a first filing in China with 24 patents, the next largest patenting country is the United States with 8 patents, as presented in Figure 1.

European countries are less active, the European Patent Office with 6 patents is the fourth on the list. Additionally, these patents are old.

**Figure 2:**
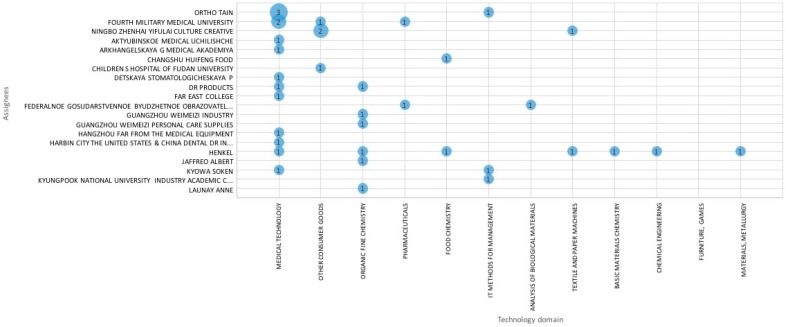
Patent families by Technology domain/Assignees. This graph represents the individual or the company that has rights and title to the invention (assignee) by technology domains. The size of the blue circles represents the number of the patent documents.

Figure 2 shows the main technology areas (at least 2 patents) protected by top applicants. Categorizations by technology domain are based on groupings of International Patent Classification (IPC) codes, so patents can appear in several different categories. There are no dominant patent assignees in the field, 20% of patent families is owned by the top 10 players, the biggest applicant is Ortho Tain with 3 patents (see Figure 2). Most patents belong to the medical technology field, there is hardly any invention in the fields of pharmaceutics or biotechnology.

### 3.2. Innovations on Prevention, Treatment and Diagnosis

The main approaches of dental caries management are the primary prevention (addressing risk factors) and secondary prevention (e.g., screening) [12] have been reflected by the patents. We examined the selected inventions according to the area they intended to function: prevention (e.g., eliminate of bad habits, oral hygiene tools), treatment (e.g., fillings, crowns) and diagnostics (e.g., remote dental examination). 37 patents were identified as prevention-related, 16 patents as treatment-related and 8 patents as diagnostics-related.

Regarding the type of inventions, the 43 specific caries management related invention are represented in all categories: prevention (23), diagnostic (4) and treatment (16) according to their function. The generic caries related supportive inventions were categorized as preventive (14) or diagnostic (3), since they intend to aid identifying early signs of future dental symptoms or help preventing the onset of future anomalies which would need extensive treatment. 12 patents from the field of orthodontics were focused on primary dentition, 11 of these orthodontic tools provide preventive conditioning to aid proper dentition and help to guide and correct early malocclusions. Since these supporting orthodontic tools are part of the preventative orthodontics for children, which are used for early corrections in the primary or early mixed dentition and intended to prevent the onset of major orthodontic anomalies, we have included them in the prevention category. One orthodontic invention was designed for orthodontic diagnostics, so we added it to the diagnostic category.

The prevention area was active throughout the years of the survey, with an increase from 2004. There was no major spike in the diagnostics field and it has been the least innovative field with 8 patents from 1997 to 2018. The treatment area became active in 2001, and its most significant year was 2016 with 5 patents.

**Figure 3:**
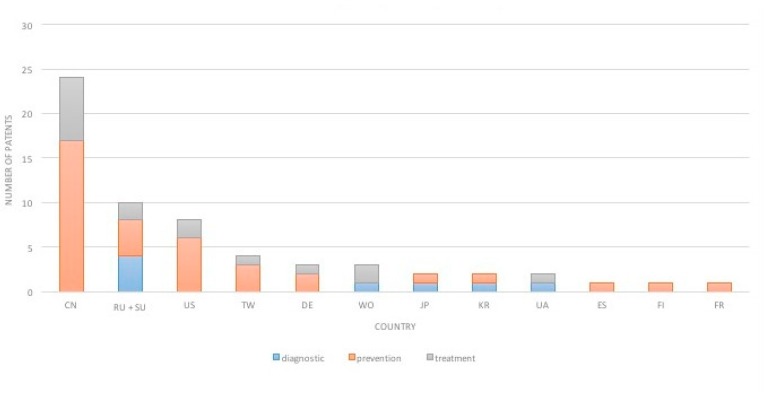
Number and type of patents per country. This graph represents the number and type (diagnostic, prevention or treatment) of patents per country. Abbreviations: CN-China, RU-Russia, SU- Union of Soviet Socialist Republic, US-United States, TW-Taiwan, DE-Germany, WO-World Intellectual Property Organization, JP-Japan, KR-Korea, Republic of, UA-Ukraine, ES- Spain, FI-Finland, FR-France.

We also examined whether various countries are specialized in individual directions (Figure 3.). China owns most of the dental prevention-related patents (17), second is the United States with 6 patents, and third is Russia (including the former Soviet Union) with 4 patents. China also has the most treatment-related patents, while Russia (including the former Soviet Union) owns the most patents among dental diagnostics-related inventions.

The prevention area is rather diverse. It includes antibacterial mouthwash with a specific protein for an antibacterial effect and children toothpastes, such as the fluoride-free, edible child nutrition toothpaste with vitamins and calcium, formulated especially for 2–5-year old children to prevent and control caries. Specifically designed toothbrushes for babies and infant oral hygiene tools, children toothbrushes and tooth protecting products like lollipops, cookies or lozenges were also inventions in this area. The tooth protecting lollipop is an invention that helps maintain balanced nutrition, control of dental caries and has a mouth-guard function. Tooth protecting cookies were patented as a baby dental care cookie, which helps to prevent tooth decay and also contains vegetables as ingredients among various flours. A large variety of orthodontic innovations created a subgroup among the prevention category with a percentage of 46%, such as dental appliances for malocclusion treatment, jaw inductors, and dentition development guide devices, such as a dentition development device to treat malocclusion.

The field of treatment contains inventions on dental restoration (e.g., excavator spoon, filling materials, colored dental filling system, dental matrix), dental prosthetic tools (e.g., crowns, absorbable endowels for deciduous teeth) and other methods and tools for tooth extraction, vital pulp extirpation method for primary teeth, cyst treatment of the primary dentition, a tooth extraction method to reduce trauma and toothache treatments with a traditional Chinese medicine formula.

The patents of the diagnostics field cover the Streptococcus mutans diagnosis method (wherein dental caries protection is provided by the use of colloidal silver), remote tele-dental systems, caries detection tools and methods to predict developmental failures. A school dental health examination tool has been patented, providing an easier way to record data about children’s teeth. It enables us to review past examination results as well and the report may be transferred to a health management system or can be printed with recommendations immediately for the children’s parents. An infrared diagnostic tool for baby tooth eruption has also been patented, it uses infrared gum thermography to identify local hyperthermia in the gum area.

## 4. Discussion

Novel techniques are crucial in dental prevention and treatment of dental caries because of its prevalence and the financial burden of the disease [13]. Novel preventive technologies, such as antimicrobial peptides, immunizations, and probiotics may reduce the disease burden on the health care system [13]. Fu Chen and Dong Wan [14] confirmed the practical value of several novel technologies for the prevention and treatment e.g., use of dental biofilm to promote the remineralization process. Another well-known example of patents is the 3D printing which was introduced over three decades ago, has gained widespread acceptance in dentistry [15].

Globally, 61 patent families were specific for children (from teeth eruption to permanent dentition) which is disproportionately low. For comparison, 23,469 patents for dental implants have been found (FamPat, search in title, abstract and claims), most of them after 2015. Another example is a study on anticancer inventions published in 2014 that identified 25,254 patent documents from year 1993 to 2013 [16]. However, the inventions addressing primary teeth caries are relatively recent, only 20% of the patents on childhood caries products have expired by 2018 worldwide.

The majority of the patents were related to prevention. This category includes caries prevention and also prevention of orthodontic anomalies. This outcome is remarkable, because caries is largely preventable by early detection [6] and early orthodontic treatments (e.g., address crowding, crossbite) can be essential to obtain normal occlusal relationship [17] and to improve children’s the oral health-related quality of life [18]. China retains its dominance, as eight of the top fifteen assignees in childhood caries prevention and treatment are based there. The European role is negligible regardless of the field of innovation (diagnostic, treatment or prevention). The lack of significant academic input into childhood caries research and the minimal number of corporate players are important indicators of poor research interest, while the absence of a collaborative environment is reflected by there being only 4 co-assigned patents.

There can be reasons behind the large differences among countries regarding the number of patents owned. Differences in R&D cultures, research interest, research focus, and location of large companies can all influence the ranking in priority countries. The quality of the granted patents can also be different. The monetary incentives of R&D can lead to higher quality inventions. More patent applications and lower quality patents can show a higher number quantitatively. The patents can also be filed and granted domestically or internationally – whether or not a country intends to protect its intellectual property abroad. The local patent laws can differ [19], thus the international evaluation could be more challenging for patents with lower quality, this might also be a reason for an increased number of domestically filed patents [20].

Limitation of this study that the patented inventions were not assessed according to their clinical usefulness and/or applicability. Although patents play an important role in the entire technology life cycle, the invention being patented can guarantee neither clinical usefulness nor success on the market. Furthermore, although our research was based on the data from the leading licensor of intellectual property, the secondary data might not cover all the patents granted globally. Additionally, several inventions are actually not granted patents at all.

The overall number of patent applications is gradually growing but such a trend is not homogenous among different medical fields. The vital question is, “why is research related to caries of primary teeth being left behind?” The most common reason for the failure in the chain of R&D is the small market size, e.g., rare diseases (because of the low number of affected patients) or neglected tropical diseases (because of paying capacity of poor patients). But beyond the small size of the market, short-sighted public policies or the imposition of taxes and price controls on related products can be barriers to the creation of new treatments as well [21]. Although such a financial barrier of R&D could be compensated by sufficient research initiatives and programs, limited effort has been devoted to this problem.

In the field of caries of deciduous teeth including ECC, as we speculate that beyond demand side barriers, supply side barriers are also present. From the supply side, the companies may be discouraged from development of new treatments for children due to the necessary ethical licenses. Caries of deciduous teeth affect children from disadvantaged and vulnerable families [22]. Although research involving children may be justified (adults have no deciduous teeth), there are always potential risks of harm. Another potential explanation is that neither the families nor the related professionals have sufficient capacity to express/protect their own interests. And unfortunately, it is the most commonly ignored chronic disease among children. Primary teeth problems are temporary, weakening the justification of involving children into clinical studies. On the other hand, children with dental caries, especially with ECC, are at increased risk for new decay in both their primary and permanent teeth [5]. Moreover, evidence suggests that potentially effective interventions against ECC should occur in the first 2 years of a child’s life [1].

## 5. Conclusions

Although the statistical data on early childhood caries is limited and vary in each country, the need for more effective treatment and multifactorial caries prevention is crucial. In our study, we examined patents related to primary dentition as well as childhood caries prevention, diagnostics and treatment inventions. The fact that there are only 61 relevant patent families available from the last 87 years reveals the research negligence of this field. Europe’s contribution to R&D in caries of the primary teeth is limited, as proved by the very few patents originating from Europe. Such a research gap should be addressed by European research policy makers and institutional research and innovation managers.

## Appendix A

List of patents included into the analysis by publication numbers

RU2661612 C1; CN108042387 A; CN107625662 A; KR20180001267 A; CN206761096U U; CN206303986U U; RU2621534 C1; CN106806028 A; CN106581159 A; TWM539272 U; CN206062509U U; CN105832854 A; WO200283022 A2; CN105795088 A; CN105662606 A; CN205268315U U; CN205215392U U; CN205144814U U; TW201603782 A; CN105266904 A; CN105169375 A; CN104430770 A; US20150050621 A1; UA--89688U U; CN103751314 A; CN202698198U U; CN202698175U U; KR20130107519 A; RU2011128892 A; CN202143714U U; ES2379170 A1; CN102188099 A; CN201692076U U; CN101889969 A; RU--86448 U1; RU2376014 C1; WO200924372 A1; US20070238071 A1; CN201070106U Y; UA--19012U U; JP2007160073 A; TWM281603 U; US7500576 B1; TWM253261 U; US20040058295 A1; US20040126743 A1; RU2204337 C2; JP2002169887 A; WO200230313 A2; DE19936461 A1; US5882192 A; FI9406191 A0; RU94028402 A; SU2072786 C1; DE9013703 U1; SU1743598 A1; US5078732 A; DE3437090 A1; SU1050697 A1; FR2143622 A1; US1816582 A
